# Editorial: Urticaria and mimickers of urticaria

**DOI:** 10.3389/falgy.2025.1625291

**Published:** 2025-05-27

**Authors:** R. Maximiliano Gomez, Beré Kezia Mahoney

**Affiliations:** ^1^ School of Health Sciences, Catholic University of Salta, Salta, Argentina; ^2^ School of Health and Wellbeing, University of Worcester, Worcester, United Kingdom

**Keywords:** allergy, diagnosis, treatment, urticaria, mimickers

**Editorial on the Research Topic**
Urticaria and mimickers of urticaria

Knowledge of urticaria has increased in recent years, but clinical variations appear that challenge the physician both in diagnosis and management of them. The contribution of the present volume is focused on the many faces of urticaria, and also on the identification of “mimickers” - those entities not belonging under its umbrella.

From the Latin American side, Prof Rojo-Gutierrez et al. provide a wide frame of syndromes and diseases including skin manifestations such as papular urticaria or vasculitis urticaria, autoinflammatory disorders and others, that present with and without urticarial signs. Relatedly, from the Asia Pacific region, Prof Fok and Katelaris present an elegant revision of different diseases presenting with related skin manifestations and other situations, such as polymorphic eruption of pregnancy. Both papers include remarkable representative images of individual conditions, giving valuable practical clinical insights into these complex conditions.

Prof Sekerel et al. continue the focus on diagnostic challenges with paediatric patients. They provide practical flows and descriptions to help with differential diagnosis in conditions presenting with urticaria and those with urticarial lesions in paediatric populations, a frequent unmet need among paediatricians and allergologists.

Prof López et al. focus on *omalizumab*, a globally accepted second step treatment implemented on refractory urticaria to anti histamines. They describe their in-depth analysis on T-cell sub-populations, proposing this evaluation as a response and follow-up strategy for patients receiving this treatment.

Finally, Prof Giustozzi et al. offer a joint vision and management of the disease between allergologists and dermatologists. They outline their evaluation of available results on the effects of phototherapy as an alternative treatment and a valuable tool to be considered in urticaria.

We have certainty these papers will provide readers with valuable but challenging information and updated reviews on a topic that demands even more knowledge if we are to better understand and manage urticaria, essential to patient-centred care.

